# Reduction, evolutionary pattern and positive selection of genes encoding formate dehydrogenase in Wood–Ljungdahl pathway of gastrointestinal acetogens suggests their adaptation to formate‐rich habitats

**DOI:** 10.1111/1758-2229.13129

**Published:** 2023-02-13

**Authors:** Ye Yao, Bo Fu, Dongfei Han, Yan Zhang, Zhiyuan Wei, He Liu

**Affiliations:** ^1^ School of Environmental and Civil Engineering, Jiangsu Key Laboratory of Anaerobic Biotechnology, Jiangsu Engineering Laboratory for Biomass Energy and Carbon Reduction Technology Jiangnan University Wuxi China; ^2^ Jiangsu Collaborative Innovation Center of Technology and Material of Water Treatment Suzhou China; ^3^ School of Environmental Science and Engineering Suzhou University of Science and Technology Suzhou People's Republic of China; ^4^ Institute of Environment and Sustainable Development in Agriculture Chinese Academy of Agricultural Sciences Beijing China; ^5^ Laboratory of Genomic and Precision Medicine, Wuxi School of Medicine Jiangnan University Wuxi Jiangsu China

## Abstract

Acetogens are anaerobes using Wood–Ljungdahl pathway (WLP) as the terminal electron acceptor for both assimilation and dissimilation of CO_2_ and widely distributed in diverse habitats. However, their habitat adaptation is often unclear. Given that bacterial genome evolution is often the result of environmental selective pressure, hereby we analysed gene copy number, phylogeny and selective pressure of genes involved in WLP within known genomes of 43 species to study the habitat adaption of gastrointestinal acetogens. The gene copy number of formate dehydrogenase (FDH) in gastrointestinal acetogens was much lower than that of non‐gastrointestinal acetogens, and in five cases, no FDH genes were found in the genomes of five gastrointestinal acetogens, but that of the other WLP genes showed no difference. The evolutionary pattern of FDH genes was significantly different from that of the other enzymes. Additionally, seven positively selected sites were only identified in the *fdhF* genes, which means *fdhF* mutations favoured their adaptation. Collectively, reduction or loss of FDH genes and their evolutionary pattern as well as positive selection in gastrointestinal acetogens indicated their adaptation to formate‐rich habitats, implying that FDH genes catalysing CO_2_ reduction to formate as the first step of methyl branch of WLP may have evolved independently.

## INTRODUCTION

Acetogens are a diverse group of anaerobes using reductive acetyl‐CoA pathway (Wood–Ljungdahl pathway [WLP]) as the terminal electron accepting process for both assimilation and dissimilation of CO_2_ (Drake, 1994; Ragsdale & Pierce, 2008; Schuchmann & Mueller, 2016). The acetogens are phylogenetically diverse, which were presented in 23 genera with more than 100 isolated species (Müller & Frerichs, 2013) and widely distributed in a variety of anaerobic habitats, including gastrointestinal tracts of humans and animals, soil, sediments, and sewage sludge (Braun & Gottschalk, 1982; Drake et al., 2008; Ljungdahl, 2009; Schnurer et al., 1996; Traunecker et al., 1991). Environmental selective pressure is an important driving force to evolution by affecting the growth and metabolism of microorganisms. Adaptation to the change of natural environment contributes to the diversity of bacterial genome (Konstantinidis et al., 2006; Konstantinidis & Tiedje, 2004). Key mutations were identified in *acsA* and *cooC* genes encoding the complex of carbon dehydrogenase/acetyl‐CoA synthase (CODH/ACS) of *Eubacterium limosum* ATCC 8486 under a high concentration of CO, indicating its adaptive laboratory evolution (Kang et al., 2020). The mutation of *ackA* gene encoding acetate kinase contributed to the improvement of acetate‐producing ability of *Sporomusa ovata* under a high concentration of H_2_‐CO_2_ (Tremblay et al., 2015).

Our previous study indicated *Clostridium bovifaecis* isolated from cow manure lacks gene encoding formate dehydrogenase (FDH), which catalyses CO_2_ reduction to formate as the first step of the methyl branch of WLP. It did not autotrophically grow on H_2_‐CO_2_ and acetogenically utilized glucose only with the supplementation of formate (formate‐dependent acetogenesis) (Yao et al., 2020). Interestingly, *Marvinbryantia formatexigens* did not grow with H_2_‐CO_2_ and homoacetogenically fermented glucose with formate (Wolin et al., 2003, 2008; Yao et al., 2020). Similarly, *Syntrophococcus sucromutans* having no FDH activity did not use H_2_‐CO_2_ and used fructose when supplied with formate (Krumholz & Bryant, 1986). The formate‐dependent acetogenic growth of the above‐mentioned two acetogens probably also be due to their lack of FDH and its encoding genes. We hypothesized that the lack of FDH genes in the genome of gastrointestinal acetogens may be associated with their adaption to formate‐rich habitats (Gomez et al., 2019; Pietzke et al., 2020), considering their isolation sources are faeces or rumen. Hereby, we studied the adaptive genome evolution of gastrointestinal acetogens, focusing on the genes involved in enzymes of WLP, especially on the genes encoding FDH. As formic acid/formate is a consolidated commodity chemical, the genetic study on FDH of acetogens is helpful for CO_2_ conversion technologies (Alpdagtas et al., 2022; Cakar et al., 2020).

Gene duplication and gene loss are important evolutionary forces, both resulting in changes of the genome or gene family size (Albalat & Canestro, 2016). The change of copy number of genes is an indicator of gene duplication or loss, which provides evidence for adaptive expansion and reduction of the genome or gene family size (Demuth & Hahn, 2009). However, no systematic investigation of genomes has been done on the gastrointestinal acetogens, especially genes involved in WLP. To date (April, 2022), 47 acetogenic species and their genomes have been reported. Given the availability of genome‐sequence information of acetogens, we used copy number, phylogenetic and selection pressure analysis of genes involved in WLP to investigate the environmental adaptation of gastrointestinal acetogens, aiming to explore the evolution of genes involved in WLP of gastrointestinal acetogens associated with their adaptation to the habitats.

## EXPERIMENTAL PROCEDURES

### Acetogen genomes and pangenome analysis

A total of 124 genomes of 47 species of acetogens belonging to 19 different genera were reported until April 2022, containing 43 complete genomes and 81 draft genomes. The genomes studied in this study were downloaded from National Center for Biotechnology Information (NCBI) database (https://www.ncbi.nlm.nih.gov/genome/). The completeness and contamination of the genomes were estimated by searching for lineage‐specific marker genes using CheckM software (Parks et al., 2015). Based upon minimum mandatory genome reporting standards of ˃90% completion, ˂5% contamination, 11 genomes were excluded, so the rest 113 genomes were included in this study (Table S1). Considering some strains without clear isolation sources and the similarity of genes involved in WLP in different strains of the same species, generally one strain within one species with clear isolation source was studied. Genomes of strains without clear isolation source were only applied for the identification of loss of certain genes.

The Bacterial Pan Genome Analysis tool (BPGA) v1.3 was employed for the pan‐genome analysis (Chaudhari et al., 2016), and protein sequences of genomes were inputted for BPGA analysis. USEARCH Clustring Algorithm (Ultra‐fast) v11.0.667 was employed to cluster genes into orthologous clusters and the value of sequence similarity cut‐off was 50%. The function annotation of pan‐genome was based on COG of Proteins database (Galperin et al., 2015; Kanehisa et al., 2004). A phylogenetic tree was constructed based on the pan‐genome of the 43 acetogenic species using neighbour joining method (NJ) in BPGA.

### Phylogenetic analysis

In order to study the evolutionary differences of genes encoding enzymes involved in WLP, three phylogenetic trees based on nucleotide sequences of genes involved in WLP were constructed using NJ method in MEGA v7.0 (Kumar et al., 2018) under *p*‐distance models with 1000 bootstraps. A NJ tree on basis of the concatenation of genes encoding enzymes involved in WLP was constructed. In addition, a NJ trees were also constructed based on the concatenation of genes encoding formate‐tetrahydrofolate (THF) ligase (FHS), methenyl‐THF cyclohydrolase (MC), methylene‐THF dehydrogenase (MD), methylene‐THF reductase (MR) and methyltransferase (MT) and carbonmonoxide dehydrogenase/acetyl‐CoA synthetase (CODH/ACS), and on genes encoding FDH, referring as FHS‐MC‐MD‐MR‐MT‐CODH/ACS tree and FDH tree, respectively.

To visualize the inconsistencies between taxonomy and phylogeny, a NJ phylogenetic tree based on nucleotide sequences of 16S rRNA gene was constructed using the RNAmmer v1.2 (Lagesen et al., 2007). Since no 16S rRNA gene sequence could be retrieved from the genome of *Acetobacterium dehalogenans* strain DSM 11527 via RNAmmer v1.2, we constructed a 16S rRNA gene phylogenetic tree of the 42 acetogenic species.

### Selective pressure analysis

To analyse selective pressure on genes encoding enzymes involved in WLP, PAML CODEML version 4.1 was employed to identify positively selection sites (Yang, 2007). The value of ω (dN/dS, the nonsynonymous/synonymous rate ratio) > 1 suggest positive selection. Nucleotide sequences of *acsA*, *acsB*, *acsC*, *acsD*, *fdhF*, *fhs*, *fchA*, *folD*, *metF*, *metV* and *acsE* were extracted from the genomes downloaded from NCBI and included in separate data sets to be analysed. Phylogenetic trees for each data set were constructed using NJ method in MEGA v7.0 under *p*‐distance models with 1000 bootstraps.

Site models allow ω values of different sites to vary and could be used to analyse the genes at the level of codon. Two pairs of site models M1a versus M2a and M7 versus M8 were applied to identify positively selection sites with *ω* > 1. In the first pair of site models, M1a (nearly neutral) model allows for the existing of two types of codon sites including conservative sites (0 < *ω*_0_ < 1) and neutral sits with (*ω*_1_ = 1); Compared with M1a, M2a (positive selection) model allows for the existing of another class of codon sites with *ω*_2_ > 1 (Nielsen & Yang, 1998; Yang et al., 2005). In the second pair of site models, M7 (beta) model assumes that the *ω* values at different codon sites falling into the range of 0 < *ω* < 1 with a beta distribution, and M8 (beta and *ω*) allows for the existence of codon sites with *ω*_2_ > 1 (Yang et al., 2000). The comparison of M1a versus M2a and M7 versus M8 are implemented with model = 0 and variable Nssites whose value for null model M1a and its alternative model M2a, null model M7 and its alternative model M8 are 1, 2, 7 and 8.

Likelihood ratio tests (LRTs) were employed to examine the hypotheses of alternative models M2a and M8 by comparing the test statistics of twice difference of the log‐likelihood values (2△*lnL*) under M1a versus M2a and M7 versus M8 with a χ^2^ distribution and degrees of freedom (df) (Yang, 1998). In LRTs for M1a versus M2a and M7 versus M8, the value of df was 2. If the LRT is significant, the Bayes empirical Bayes (BEB) was used to identify positively selected sites by calculating the posterior probabilities for sites (Yang et al., 2005). Amino acid site with BEB posterior probability >95% were identified as positively selected sites.

### Identification of difference in category and copy number of genes

The difference in category and copy number of genes encoding enzymes involved in WLP was identified via the comparison of genomes data among 43 acetogenic species downloaded from NCBI. Gene copy number in this study refers to the number of genes encoding an enzyme rather than the number of times it was copied during quantitative PCR (Demuth & Hahn, 2009; Redon et al., 2006). In order to reduce the likelihood of false negatives, for the genes absent in some bacterial genomes, BLAST on NCBI was used to compare the nucleotide and protein sequences of related strains against these genomes lacking the genes (Boratyn et al., 2013). Statistical analysis of the difference of gene copy number was performed using the unpaired *t*‐test with GraphPad Prism 9.2.0.

## RESULTS

### Genomic features

More than 100 acetogenic bacteria have been isolated from diverse habitats. A total of 113 genomes of 43 acetogenic species were available for this study, which included 74 gastrointestinal strains and 39 non‐gastrointestinal ones. A summary of features of these genome sequences is listed in Table S1. The completeness of all the studied genomes were more than 90%, and the contamination were less than 5%. The G + C contents of these genomes ranged from 28.4% to 62.9% and their sizes ranged from 2.40 to 7.09 Mb.

### Pangenome analysis

**FIGURE 1:**
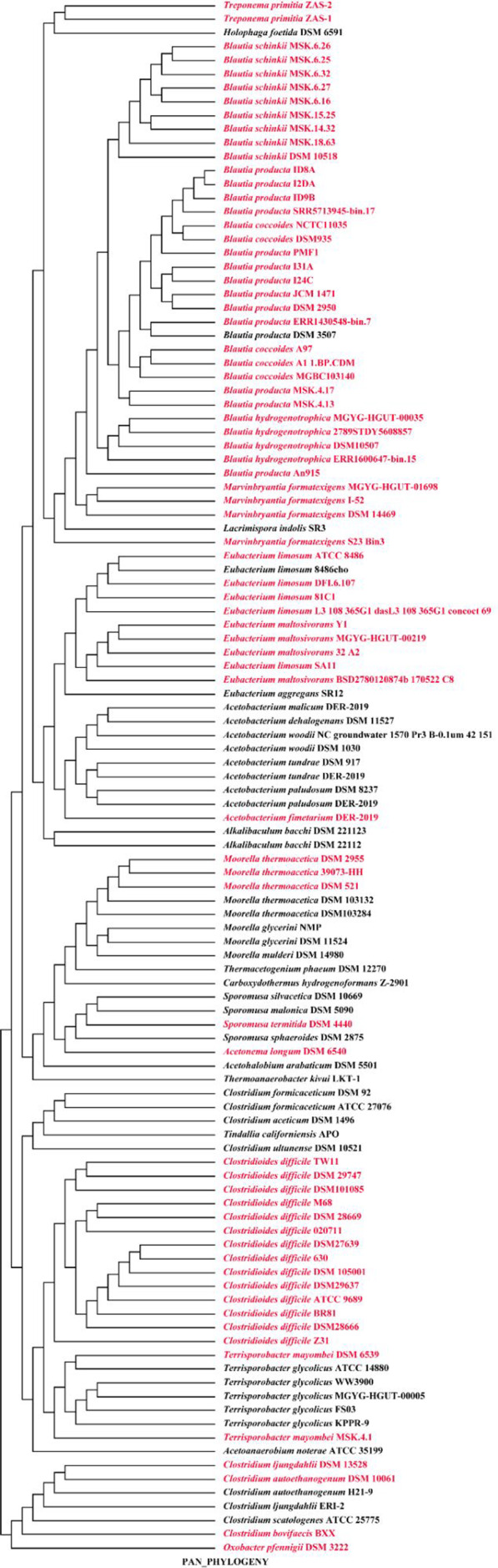
Neighbour‐joining phylogenetic tree based on pan‐genome of the 113 strains of the 43 acetogen species. Red and black fonts represent gastrointestinal and non‐gastrointestinal acetogenic strains, respectively.

To understand the evolutionary relationship among these acetogenic strains, a phylogenetic tree based on pan‐genome (Figure 1) and 16S rRNA gene was constructed (Figure S1). The pan‐genome tree showed that strains were generally clustered by the same species or same genera (Figure 1). A total of 113 strains were assigned to two clades: a large clade mainly consisting of *Treponema Primitia*, *Blautia* species, *M. formatexigens*, *Acetobacterium* species, *Eubacterium* species, *Clostridium* species, *Moorella* species, two *Terrisporobacter* species, *Sporomusa* species and *Clostridioides difficile*; and a small clade consists of six strains of *Clostridium* spp. and *Oxobacter pfennigii* DSM 3222. Unexpectedly, *C. ljungdahlii*, *C. autoethanogenum*, *C. bovifaecis* and *C. scatologenes* clustered outside of the main clade containing *C. formicaceticum*, *C. carboxidivorans* and *C. ultunense*, suggesting the gene composition of the four *Clostridium* species were not similar with the other three species, and exhibited the least amount of evolutionary change from a common ancestor. The comparison between the pan‐genome tree and the 16S rRNA tree also shows that phylogenetically closely related organisms show a higher evolutionary closeness of pan‐genome (Figure S1).

To reveal the genomic features of studied acetogens, BPGA tool (Chaudhari et al., 2016) was employed to identify orthologous groups among the genomes. A total of 411,499 genes were identified in the pan‐genome. Of these genes, 565 genes (0.14%) were clustered into the core genome (shared by all strains), 362,0394 (87.98%) genes were represented in the accessory genome (existing in two or more strains), and 48,895 (11.88%) genes were identified as unique genes (unique to single strain). The low percentage of core genome is expected since most known acetogens are phylogenetically and metabolically diverse bacteria present in 23 different genera with a central metabolic WLP.

**FIGURE 2:**
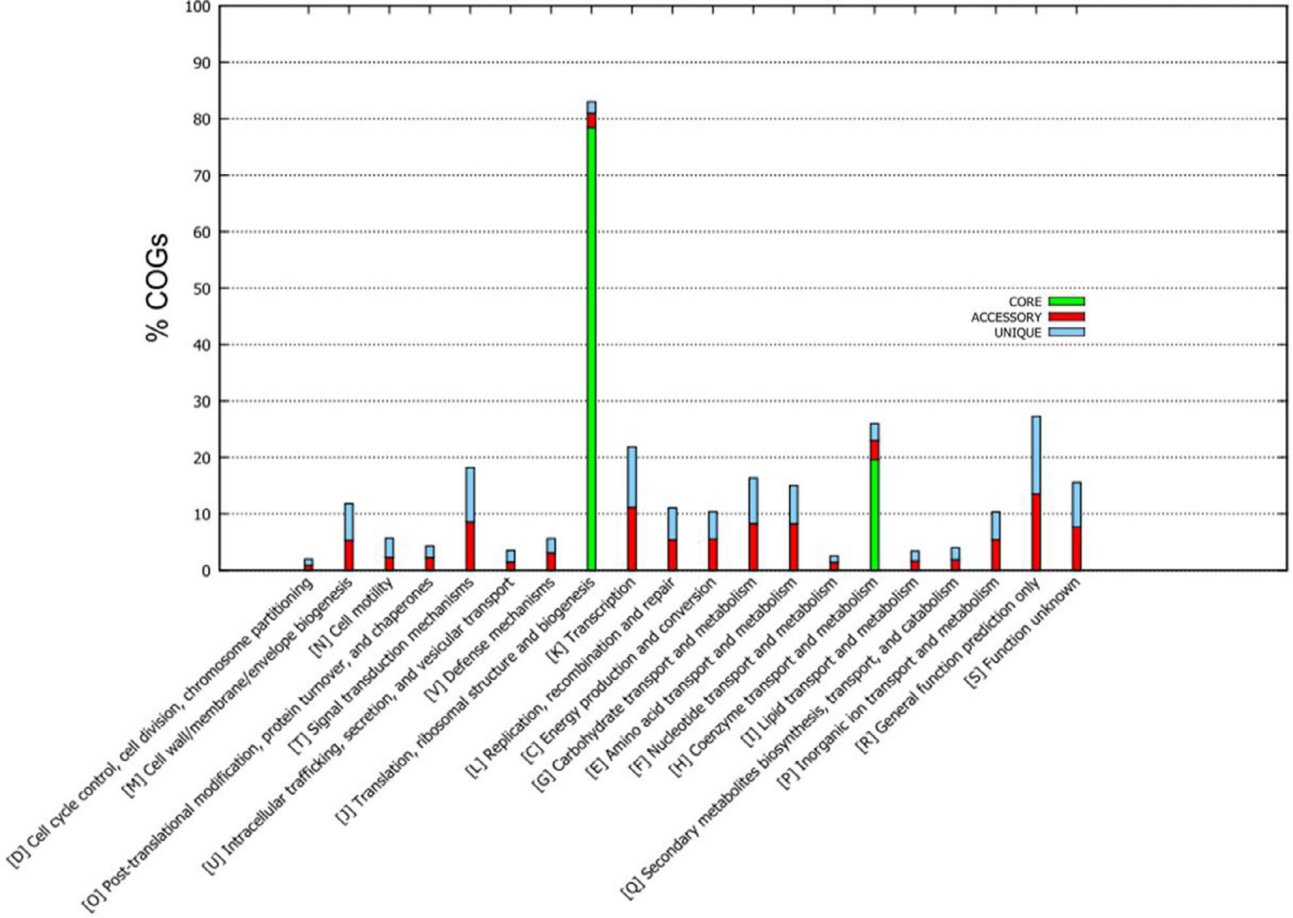
Function annotation of pan‐genome of the 113 strains of 43 species of acetogens based on Cluster of Orthologous Group of Proteins (COG) database. Green, red and blue columns represent core, accessory and unique genes in the pangenome.

Protein sequences from core, accessory, and unique genome were classified with the Clusters of Orthologous Groups (COG) database. The core genome had the highest percentage of sequences from translation, ribosomal structure and biogenesis (category J, 80%), and coenzyme transport and metabolism (category H, 20%) (Figure 2). The highest percentages of sequences in the accessory and unique genomes were from transcription (category K, 12.83% and 12.59%, respectively) and general function prediction only (category R, 15.60% and 16.07%, respectively) (Figure 2). The third most represented categories for the accessory and unique gene families were category T (signal transduction mechanisms, 9.85% and 11.30%, respectively).

### The copy number of genes involved in WLP

**FIGURE 3:**
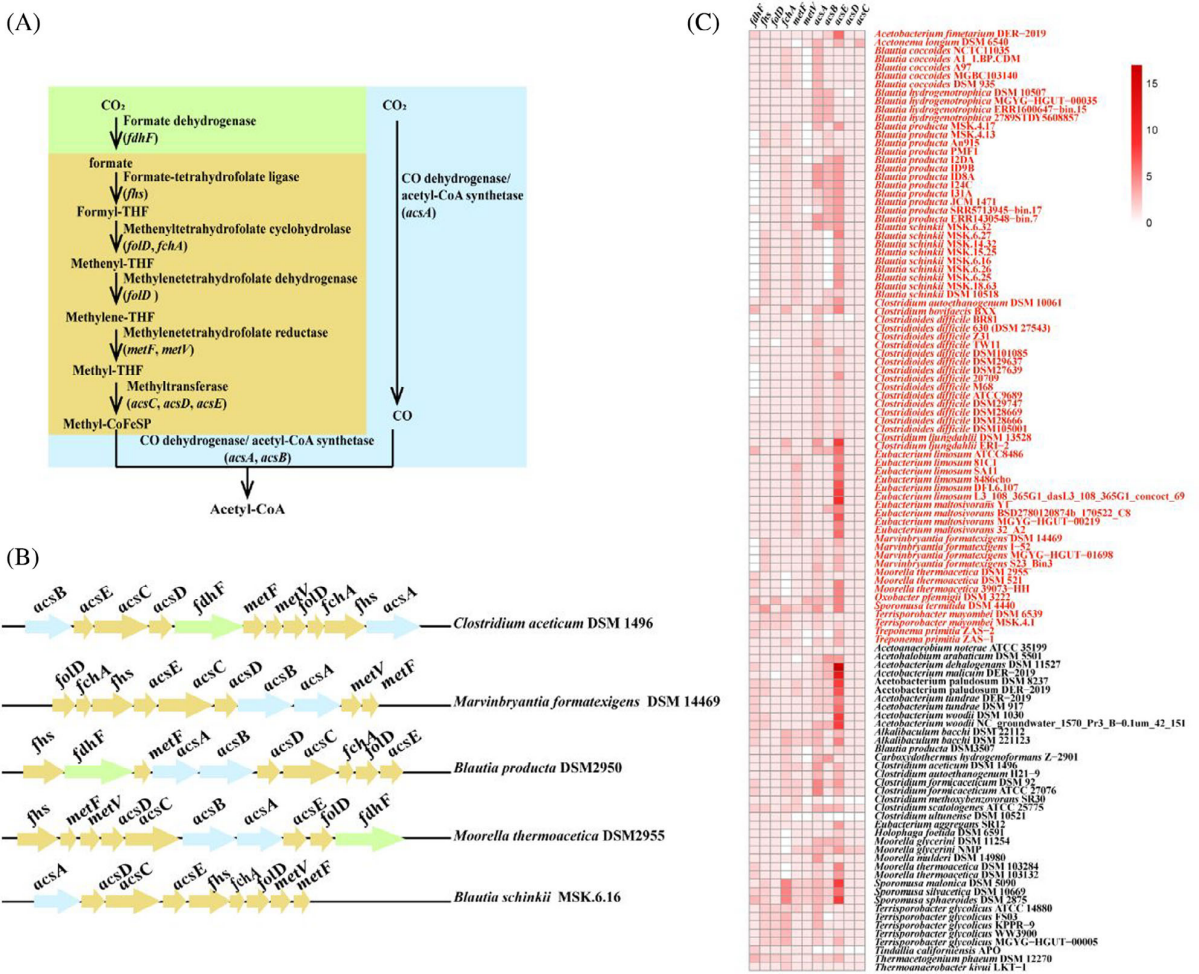
Genes encoding enzymes involved in Wood–Ljungdahl pathway (WLP) in the genomes of 43 acetogenic species. Green, yellow and blue represents genes encoding formate dehydrogenase, and genes encoding formate‐tetrahydrofolate ligase, methenyltetrahydrofolate cyclohydrolase, methylenetetrahydrofolate dehydrogenase, methylenetetrahydrofolate reductase and methyltransferase, as well as genes encoding carbon monoxide dehydrogenase/acetyl‐CoA synthetase (A, B); copy number of genes encoding enzymes involved in WLP (C). Red and black fonts represent gastrointestinal and non‐gastrointestinal acetogens, respectively.

To gain a better appreciation for the genome evolution of gastrointestinal acetogens, we performed copy number statistics of genes encoding seven enzymes of WLP including FDH, formate‐tetrahydrofolate (THF) ligase (FHS), methenyl‐THF cyclohydrolase (MC), methylene‐THF dehydrogenase (MD), methylene‐THF reductase (MR), methyltransferase (MT) and carbon monoxide dehydrogenase/acetyl‐CoA synthetase (CODH/ACS), aiming to identify the differences in the copy number of genes responsible for WLP between gastrointestinal and non‐gastrointestinal acetogens (Figure 3A). It should be noted that copy number of gene or gene copy number in this study was referred to the number of genes encoding subunits of an enzyme in a bacterial genome (Demuth & Hahn, 2009; Redon et al., 2006). Based on the presence of genes encoding seven enzymes of WLP, there are 15 types of WLP genes in the studied genomes with *C. aceticum* DSM 1496, *M. formatexigens* DSM 14469, and *B. producta* DSM 2950*, B. schinkii* MSK.6.16 and *Moorella thermoacetica* DSM 2955 as the representatives for the Top 5 type (Figure 3B). For more details of WLP genes arrangement in acetogens, we refer readers to the excellent studies by Poehlein et al. (2015) and Ross et al. (2020).

**FIGURE 4:**
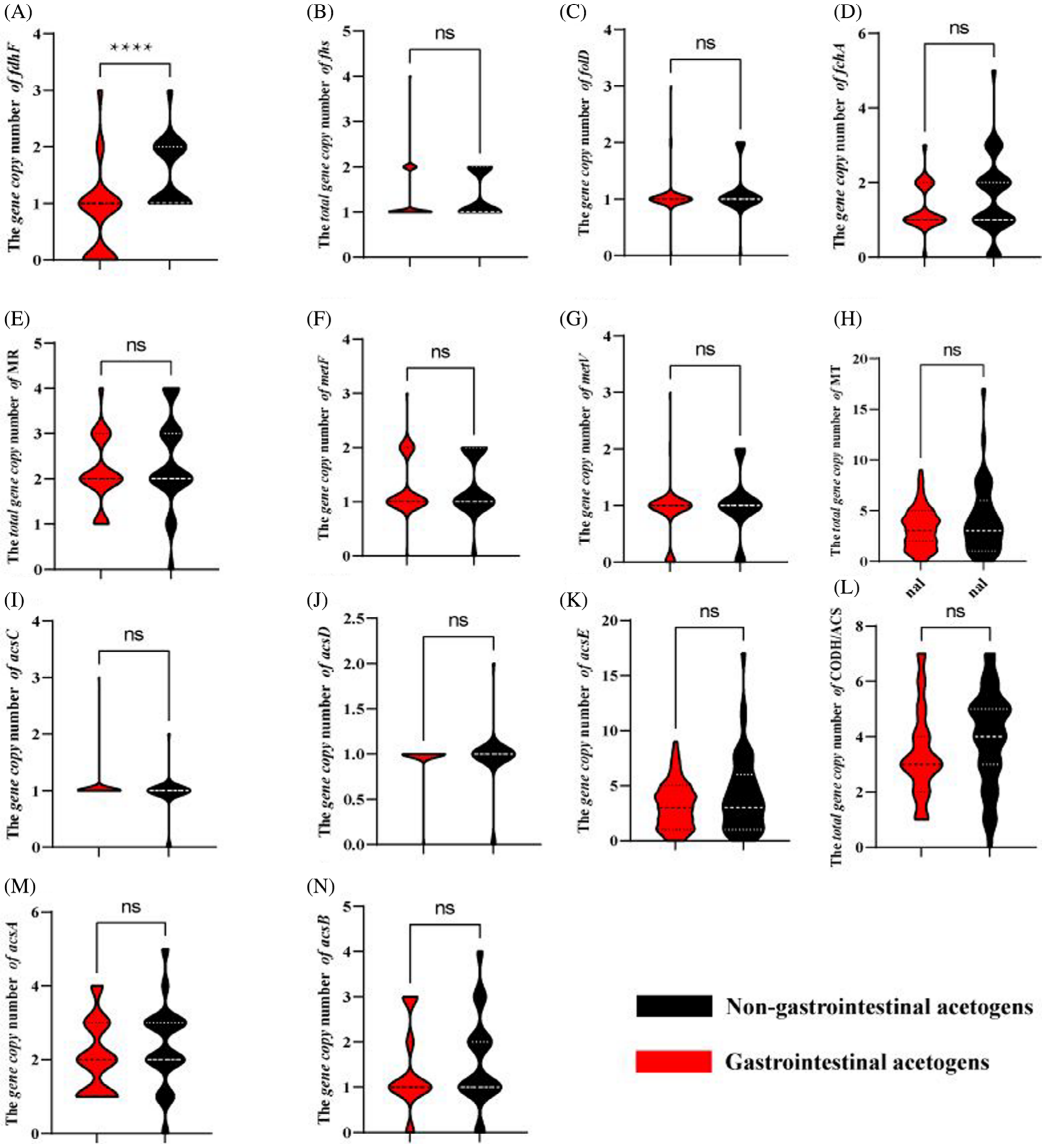
The copy number of formate dehydrogenase subunit *fdhF* (A); the copy number of formate‐tetrahydrofolate ligase subunit *fhs* (B); the copy number of *folD* (C) encoding bifunctional 5,10‐MD/5,10‐MC and methenyltetrahydrofolate cyclohydrolase subunit *fchA* (D); the total copy number of genes encoding methylenetetrahydrofolate reductase (E) and its subnutis *metF* (F), *metV* (G); the total copy number of genes encoding methyltransferase (H) and its subunits *acsC* (I), *acsD* (J) and *acsE* (K); the total copy number of genes encoding carbon monoxide dehydrogenase/acetyl‐CoA synthase (L) and its subunits *acsA* (M), *acsB* (N) (****p < 0.01; ns, *p* > 0.05). CODH/ACS, carbon monoxide dehydrogenase/acetyl‐CoA synthase; FDH, formate dehydrogenase; FHS, formate‐tetrahydrofolate ligase; MC, methenyltetrahydrofolate cyclohydrolase; MD, methylenetetrahydrofolate dehydrogenase; MR, methylenetetrahydrofolate reductase; MT, methyltransferase. Red and black fonts represent acetogens isolated from gastrointestinal and non‐gastrointestinal tracts, respectively. The width of the plots represents the amount of data for a value. The dotted line from top to bottom represents the upper quartile, median, and lower quartile, respectively.

The *fdhF* subunit of the formate dehydrogenase encodes for FDH, which catalyses the reduction of CO_2_ to formate, the first step of the methyl branch of the WLP (Figure 3A,B). As expected, the copy numbers of *fdhF* genes in gastrointestinal acetogens (average 0.7 gene copies) were significantly lower than that of non‐gastrointestinal acetogens (average 1.5 gene copies) (*p* ˂ 0.01) (Figure 4A), and even no known *fdhF* genes were found in the species or strains of gastrointestinal *C. bovifaecis*, *M. formatexigens*, *B. producta, B. schinkii* (formerly known as *Ruminococcus schinkii*) (Liu et al., 2008) and *Cl. difficile* (formerly *Clostridium difficile*) (Lawson et al., 2016) (Figure 3C, Table S2). The lack of a gene encoding FDH in *C. bovifaecis* was verified by PCR, reverse transcription‐PCR analysis, enzyme activity assay, and its formate‐dependent acetogenic utilization of glucose on DNA, RNA, protein, and phenotype level, respectively (Yao et al., 2020). A recent paper reported that only 127 isolates (44% of isolates) encode formate dehydrogenase in the 286 genomes of *Blautia* acetogenic species (Trischler et al., 2022). This finding is in accordance with the study of Shin et al., in which a number of *fdh* gene copies were different in the 14 genomes of acetogens (Shin et al., 2016).

Subsequently, formate is converted to methyl‐CoFeSP catalysed by five enzymes of the methyl branch of WLP (Figure 3A). Genes encoding formate‐THF ligase (*fhs*) converting formate to formyl‐THF were presented in all the genomes of studied acetogens (Figure 3C, Table S2). Formyl‐THF is converted to methenyl‐THF catalysed by methenyl‐THF cyclohydrolase (*fchA*) and then to methylene‐THF by methylene‐THF dehydrogenase, or the two reactions are catalysed by bifunctional 5,10‐methylene‐THF dehydrogenase/5,10‐methenyl‐THF cyclohydrolase (*folD*) (Figure 3A). Most acetogens contained both *fchA* and *folD* (Figure 3C, Table S2). Consistently with *fhs*, the copy number of *folD* and *fchA* did not differ between gastrointestinal and non‐gastrointestinal acetogens (*p* ˃ 0.05) (Figure 4B–D).

Next, methylene‐THF is converted to methyl‐THF catalysed by MR, and *metF* and *metV* encoding this enzyme were present in most acetogens, but some acetogens contained either of the genes, for example, *B. producta* DSM 2950 (formerly known as *Ruminococcus productus*) (Liu et al., 2008) containing only *metF* (Figure 3C, Table S2). The last reaction of methyl branch of WLP is conversion of methylene‐THF to methyl‐CoFeSP catalysed by MT, being encoded by *acsC*, *acsD*, and *acsE* (Figure 3A). Furthermore, the copy number of the genes encoding MR and MT showed no significant differences between gastrointestinal and non‐gastrointestinal acetogens (Figure 4E–K) (*p* ˃ 0.05). Interestingly, genes encoding MR and MT were not found in the complete genome of *C. ultunense* (Figure 3C, Table S2). This is consistent with the study of Manzoor et al., in which authors speculated there were maybe other unknown genes encoding this two enzymes (Manzoor et al., 2018).

In the carbonyl‐branch of WLP, CO_2_ reduction to CO and synthesis of acetyl‐CoA from methyl‐corrinoid [Fe‐S] protein (methyl‐CoFeSP) and CO are both catalysed by carbon monoxide dehydrogenase/acetyl‐CoA synthase complex (CODH/ACS) (Figure 3A). Genes encoding CODH/ACS in acetogens mainly included *acsA* and *acsB* (Figure 3C, Table S2), and their copies number had no difference between gastrointestinal and non‐gastrointestinal acetogens (*p* ˃ 0.05) (Figure 4L–N). Considering that *cooC* and *acsF* encode homologous accessory proteins for nickel insertion and do not perform the function of oxidoreductase (Adam et al., 2018), the two genes were not included in this study.

Overall, the total copy number of genes encoding FDH in gastrointestinal acetogens was lower than that in non‐gastrointestinal acetogens, but the copy number of the other genes involved in WLP showed no difference with statistical significance. The results indicated that reduction or complete loss of genes encoding FDH occurred in gastrointestinal acetogens.

### Phylogenetic analyses based on genes involved in WLP

**FIGURE 5:**
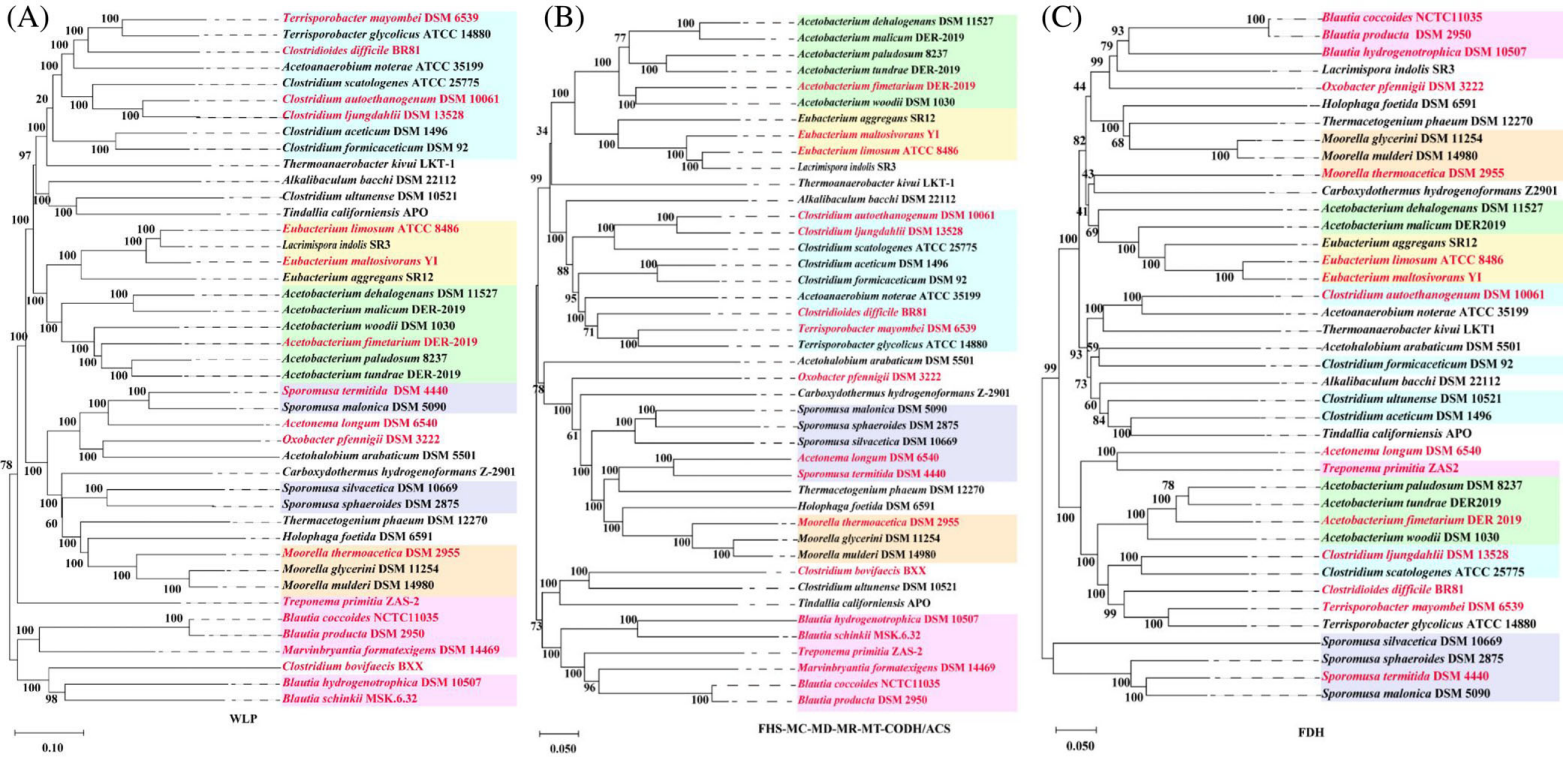
Neighbour‐joining phylogenetic tree based on genes involved in Wood–Ljungdahl pathway (WLP) (A), genes encoding formate‐tetrahydrofolate ligase, methenyltetrahydrofolate cyclohydrolase, methenyltetrahydrofolate dehydrogenase, methylene‐THF reductase, methyltransferase and carbon monoxide dehydrogenase/acetyl‐CoA synthetase (B) and based on genes encoding formate dehydrogenase (C). Bootstrap values are based on a total of 1000 bootstrap replicates. Red and black fonts represent gastrointestinal and non‐gastrointestinal acetogens, respectively.

As WLP is a metabolic feature of acetogens, a phylogenetic analysis based on genes referred to WLP will give insights into evolutionary relationship of gastrointestinal acetogens during habitat adaption. The WLP tree indicated that 43 species were assigned to two clades: the large clade mainly included five groups (see colour shading in Figure 5A), and the other clade contained four *Blautia* species, *C. bovifaecis* and *M. formatexigens* (see pink shading in Figure 5A). The evolutionary closeness based on WLP was less affected by their phylogenetic similarity in comparison with based on the pan‐genome. Four *Sporomusa* species forms two separated groups (see purple shading in Figure 5A). *C. bovifaecis* instead of *Lacrimispora indolis* (formerly known as *Clostridium methoxybenzovorans*) (Kobayashi et al., 2021) formed a cluster with *B. coccoides* (formerly known as *Clostridium coccoides*) (Liu et al., 2008), *B. schinkii*, *B. hydrogenotrophica, B. producta* and *M. formatexigens*. Interestingly, the above six species were all from mouse faeces or human faeces (Table S1) and there were many FDH‐lacking strains of *B. schinkii*, *M. formatexigens*, *C. bovifaecis* and *B. producta* (Figure 3C and Table S2). It is shown gastrointestinal acetogens from similar habitats shows relatively closer evolutionary relationship in genes referred to WLP.

Significant difference of gene copy number between gastrointestinal and non‐gastrointestinal acetogens only occurred in genes encoding FDH, suggesting that FDH genes may evolve differently from genes encoding the other enzymes involved in WLP. Thus, we constructed two phylogenetic trees based on genes encoding FHS, MC, MD, MR, MT, CODH/ACS (FHS‐MC‐MD‐MR‐MT‐CODH/ACS tree) and genes encoding FDH (FDH tree) (Figure 5B,C), respectively. The FHS‐MC‐MD‐MR‐MT‐CODH/ACS tree indicated that the 43 acetogenic species were assigned to two clades: a clade mainly consisting of *Acetobacterium*, *Eubacterium*, *Clostridium*, *Sporomusa* and *Moorella* species, a second of two *Clostridium* species, four *Blautia* species, *M. formatexigens* and *Treponema primitia* (see pink shading in Figure 5B). Both the WLP tree and FHS‐MC‐MD‐MR‐MT‐CODH/ACS tree show the cluster of *C. bovifaecis*, four *Blautia* species and *M. formatexigens* exhibited the least amount of evolutionary change from a common ancestor in terms of WLP genes.

The FDH tree was assigned to a large clade and a little one only consisted of four *Sporomusa* species (see purple shading in Figure 5C). The main clade included two subclades: a subclade mainly containing four *Acetobacterium* species, two *Clostridium* species, and two *Terrisporobacter* species; the subclade of *Blautia*, *Moorella*, *Eubacterium*, two *Acetobacterium* species and two *Clostridium* species (Figure 5C). Six *Acetobacterium* species and five *Clostridium* species were clustered in the FHS‐MC‐MD‐MR‐MT‐CODH/ACS tree but dispersed into different subclades in the FDH tree. Obviously, the clustering patterns in the FDH tree were highly inconsistent with that in the FHS‐MC‐MD‐MR‐MT‐CODH/ACS tree, which was more similar to that in the WLP tree.

The finding is consistent with the study of Ross et al. on genomic features of the *Acetobacterium* genus, in which they found that the gene cluster encodes enzymes responsible for the conversion of formate to methyl‐THF (methyl branches) and cluster (carbonyl branch) encodes the CODH/ACS complex, methyltransferase and accessory proteins for conversion of methyl‐THF to acetyl‐CoA are highly conserved across all sequenced *Acetobacterium* genomes, but cluster encoding the FDH was markedly different and more divergent in the remaining genomes, with low sequence identity (50%) and similarity (Ross et al., 2020). Combined with the reduction or complete loss of genes encoding FDH in gastrointestinal acetogens, the phylogenetic analysis implied that FDH genes evolved independently from the genes encoding the other enzymes associated with WLP.

### Assessment of positive pressure on genes encoding FDH in acetogens

To better understand whether genes encoding enzymes involved in WLP of gastrointestinal acetogens were subjected to natural selection during the adaptation to gastrointestinal tracts, site models in the PAML were employed for the identification of amino acid sites under positive selection (Yang, 2007). Considering *fdhF*, *fhs*, *fchA*, *folD*, *metF*, *metV*, *acsA*, *acsB*, *acsC*, *acsD* and *acsE* were present in the most acetogens, these genes were included in separate data sets to be analysed.

The comparisons of M1a versus M2a model for all the genes showed no difference with statistical significance (Table S3), but the comparison between M7 and M8 model for *fdhF* gene yielded a likelihood test ratio statistic of 24.5028 (*p* = 0.0000), allowing for rejection of the hypothesis of null model (M7). The comparison using M1a versus M2a model and M7 versus M8 model showed that all the genes except for *fdhF* did not display significant differences (Table 1). Bayes empirical Bayes (BEB) analysis showed that four amino acid sites with BEB posterior probability >95% and three sites with the value >99% were identified in *fdhF* as positively selected sites (Table 2). Contrastingly, no positively selected sites were identified in all the other genes encoding enzymes involved in WLP. A total of seven positively selected sites in *fdhF* gene indicated that mutations of *fdhF* were under positive selection pressure, which means favouring the growth of individuals to better fit the habitats.

**TABLE 1:** Likelihood ratio tests of the site model M7 versus M8 for *fdhF*, *fhs*, *fchA*, *folD*, *metF*, *metV*, *ascE*, *acsA*, *acsB*, *acsC* and *acsD* of in the genomes of 43 acetogenic bacteria

Gene	Model	np	(ln *L*)	2 Δln *L*	*p*
*fdhF*	M7	84	−90499.5935	24.5028	0.0000
	M8	86	−90487.3421		
*fhs*	M7	96	−38237.2238	1.8035	0.4058
	M8	98	−38238.1256		
*fchA*	M7	90	−17856.3349	0.1506	0.9276
	M8	92	−17856.2596		
*folD*	M7	90	−21893.2564	0.2174	0.8970
	M8	92	−21893.3651		
*metF*	M7	86	−20569.4432	1.0866	0.5808
	M8	88	−20569.9865		
*metV*	M7	80	−14563.2597	1.0768	0.5836
	M8	82	−14563.7981		
*ascE*	M7	90	−24599.3765	2.6294	0.2686
	M8	92	−24598.0618		
*acsB*	M7	94	−62596.4545	1.0624	0.5879
	M8	96	−62596.9857		
*acsA*	M7	86	−27596.3214	4.0218	0.1339
	M8	88	‐‐27594.3105		
*acsC*	M7	88	−24796.2567	2.6842	0.2613
	M8	90	‐‐24797.5988		
*acsD*	M7	86	−27896.3257	0.1456	0.9298
	M8	88	−27896.3985		

Abbreviations: np, number of parameters; ln *L*, likelihood value.

**TABLE 2:** Bayes empirical bayes (BEB) analysis of positively selected sites identified *fdhF* of acetogens by site model

Model	Codon	Amino acid	Posterior probability	Post mean ± SE for *ω*
M7 versus M8	12	M	0.749	1.299 ± 0.192
	44	L	0.961*	1.913 ± 0.326
	62	D	0.991**	2.386 ± 0.757
	68	Q	0.620	1.401 ± 0.478
	94	L	0.998**	2.498 ± 0.037
	109	M	0.969*	2.346 ± 0.196
	165	Q	0.986*	2.378 ± 0.183
	592	D	0.967*	2.198 ± 0.147
	629	P	0.997**	2.398 ± 0.058

* Posterior probability of BEB analysis >95%.

** Posterior probability of BEB analysis >99%.

## DISCUSSION

The pan‐genome analysis constructed a framework for estimating genome diversity and identifying the core, accessory, and unique genome before the analysis based on WLP genes. COG analysis indicated that the core genome was significantly related to a number of essential cellular functions in most bacteria (Shin et al., 2016). The most linked functional groups in the accessory and unique genomes were assigned to general function and transcription. This result and the low percentage of core genome is in agreement with the phylogenetic and metabolic diversity of acetogen present in 19 different genera as shown in the pangenome‐based phylogenetic tree, but they all possess a central metabolic WLP.

The copy number of genes encoding FDH in gastrointestinal acetogens were much lower than that in non‐gastrointestinal acetogens. Known *fdhF* genes were absent in two gastrointestinal species (*C. bovifaecis*, *M. formatexigens*) and several strains of gastrointestinal *B. producta*, *B. schinkii*, and *Cl. difficile*. The result suggested that gastrointestinal acetogens undergo similar environmental selective pressure. Natural selection pressure appears to drive the reductive evolution of bacterial genomes (Hemme et al., 2016; Zhang et al., 2017). Both nature and laboratory experiments offer a lot of examples of genome reduction due to natural selection pressure. Parasitic *Lactobacillus* spp. lost the genes for synthesizing certain amino acids, which can be provided by their host (Callanan et al., 2008; van de Guchte et al., 2006). Accordantly, seven positively selected sites were only identified in *fdhF* (Table 2), indicating that *fdhF* mutation favoured bacterial growth. Collectively, reduction or complete loss of genes encoding FDH occurred in gastrointestinal acetogens that favoured a better fit to their habitation.

The reduction or complete loss of FDH genes in gastrointestinal acetogens may be explained by the Black Queen Hypothesis (BQH), a novel theory of reductive evolution (Morris et al., 2012). Adaptive gene loss provides a selective advantage by conserving microorganism's limiting resources, provided the gene's function is dispensable (Morris et al., 2012). Given formate is rich in gastrointestinal tracts of animals (Gomez et al., 2019), the conversion of CO_2_ to formate catalysed by FDH seems to be dispensable for gastrointestinal acetogens, and the simplification of formate formation from CO_2_ can save energy (two reducing equivalents) for cell growth, anyway, the reduction or loss of FDH genes favoured the individuals in formate‐rich habitats.

Differently with the other genes involved in WLP, there was a significant reduction only in the copy number of genes encoding FDH in the gastrointestinal acetogens. The phylogenetic analysis based on the WLP and FDH genes showed that the evolutionary pattern of genes encoding FDH was significantly different from that of the other enzymes. While seven positively selected sites were identified for *fdhF*, no positively selected sites were identified in all the other genes involved in WLP. All the results implied that the evolution of FDH genes seems to be independent from the other genes. Apart from different evolutionary pattern, lower gene copy number and higher genetic diversity, FDH in acetogens catalyses the reduction of CO_2_ to formate in three manners. One is as a standalone NADP^+^‐dependent FDH for acetogenic bacteria such as *M. thermoacetica* (Schuchmann & Mueller, 2014). The second is as a subunit of hydrogen‐dependent carbon dioxide reductase (HDCR), a newly reported enzyme complex composed of an FDH (*fdhF1/2*) and an iron–iron hydrogenase (*hydA2*) in *Acetobacterium woodii* and *Thermoanaerobacter kivui* (Ceccaldi et al., 2017; Schuchmann & Muller, 2013). The third is in complex with an electron‐bifurcating [FeFe] hydrogenase which might use either H_2_ or ferredoxin and NADP^+^ as the electron donors in acetogenic bacteria such as *C. ljungdahlii* and *C. autoethanogenum* (Cakar et al., 2018; Schuchmann & Mueller, 2014; Wang et al., 2013).

Previous study of Shin et al. and Ross et al. indicated that WLP was functionally separated into three core groups: reduction of CO_2_ to formate, formation of acetyl‐CoA from the methyl‐branch and carbonyl‐branch, and acetate production from acetyl‐CoA (Ross et al., 2020; Shin et al., 2016). Jain et al. reported that one may speculate that WLP have evolved as three independent parts: CO_2_ reduction to formate, formate reduction to a methyl group and the CODH/ACS reaction (Jain et al., 2020). Combined with this study, it is suggested that the reduction of CO_2_ to formate catalysed by FDH is a functionally independent module of WLP, which is associated with its independent gene evolution from the other genes related to WLP.

The reductive evolution of FDH genes in gastrointestinal acetogens would proceed during the environmental selective pressure, which may lead to loss of FDH gene in more acetogens to be found. For the *fdh*‐lacking acetogens, for example, *C. bovifaecis* and *M. formatexigens*, they cannot grow with H_2_‐CO_2_ and acetogenically fermented glucose only with the supplementation of formate (formate‐dependent acetogenesis) (Wolin et al., 2003, 2008; Yao et al., 2020), thus 1 mol of formate and 1 mol of CO_2_ instead of 2 mols of CO_2_ are the electron acceptors for WLP during heterotrophic acetogenesis (Yao et al., 2020).

Therefore, more studies of the coupled formate + CO_2_ respiration in heterotrophic acetogenesis of FDH‐lacking acetogens would be interesting. Future studies could be performed to address on the possibility of coupled formate + CO_2_ respiration in heterotrophic acetogenesis of FDH‐containing acetogens. Considering that FDH‐lacking acetogen also fulfils the requirements of the term acetogen, once more evidence is identified to confirm the coupled formate + CO_2_ respiration in heterotrophic acetogenesis, the definition of acetogen should be complemented to contain the FDH‐lacking acetogens, which cannot grow on H_2_ + CO_2_ but perform heterotrophic acetogenesis in presence of formate + CO_2_. Additionally, another interest is the ecological role of the reductive evolution of FDH gene in gastrointestinal acetogens, particularly the relationships between FDH‐lacking acetogens and its formate‐producing helpers. Evolution in accordance with the BQH generates the dependency of *fdh*‐lacking gastrointestinal acetogens on the coexistence of formate‐producing microbes for the loss of metabolic function.

## CONFLICT OF INTEREST

The authors have no conflict of interest to declare.

## Supporting information


**Appendix S1:** Supporting Information

## Data Availability

Nucleotide sequence data reported or referred in this paper is available in GenBank database.
