# Supplementary material for: Reduction, evolutionary pattern and positive selection of genes encoding formate dehydrogenase in Wood–Ljungdahl pathway of gastrointestinal acetogens suggests their adaptation to formate‐rich habitats
Source: Environ Microbiol Rep. 2023 Feb 13;15(2):129–41. doi: 10.1111/1758-2229.13129 (PMC10103890; doi:10.1111/1758-2229.13129)
Supplement: Supplementary file 1 — Appendix S1: Supporting Information [file EMI4-15-129-s001.docx]

**Reduction, evolutionary pattern and positive selection of genes encoding formate dehydrogenase in Wood-Ljungdahl pathway of gastrointestinal acetogens suggests their adaptation to formate-rich habitats**

Ye Yao^a^, Bo Fu^a, b*^, Dongfei Han^c, d^, Yan Zhang^a, b^, Zhiyuan Wei^e^, He Liu^a, b^

^a^ School of Environmental and Civil Engineering, Jiangsu Key Laboratory of Anaerobic Biotechnology, Jiangsu Engineering Laboratory for Biomass Energy and Carbon Reduction Technology, Jiangnan University, Wuxi, China.

^b^ Jiangsu Collaborative Innovation Center of Technology and Material of Water Treatment, Suzhou, China.

^c^ Institute of Environment and Sustainable Development in Agriculture, Chinese Academy of Agricultural Sciences, Beijing, China.

^d^ School of Environmental Science and Engineering, Suzhou University of Science and Technology, Suzhou 215009, PR China

^e^ Laboratory of Genomic and Precision Medicine, Wuxi School of Medicine, Jiangnan University, Wuxi, Jiangsu, China.

*Corresponding: Bo Fu, [fubo@jiangnan.edu.cn](mailto:fubo@jiangnan.edu.cn)

**Supplemental Tables (S1-S3)**

**Table S1** General features of bacterial genomes used in this study

| **No** | **Species** | **Isolation sources** | **Strain** | **GenBank accession number** | **Assembly Level** | **Comple-teness (%)** | **Conta-mination (%)** | **Size (Mb)** | **GC (%)** |
| --- | --- | --- | --- | --- | --- | --- | --- | --- | --- |
| **Gastrointestinal acetogens** | | | | | | | | | |
|  | *Acetobacterium fimetarium* | Cattle manure | DER-2019*** | GCA_014284475.1 | Contig | 98.59 | 1.64 | 3.20 | 44.7 |
|  | *Acetonema longum* | Wood-eating termite gut | DSM 6540*** | GCA_000219125.2 | Scaffold | 99.37 | 3.16 | 4.30 | 50.4 |
|  | *Blautia coccoides* | Mouse feces | NCTC11035*** | GCA_900461125.1 | Contig | 99.36 | 0.00 | 6.10 | 45.6 |
|  |  | Mouse feces | A1_1.BP.CDM | GCA_910573705.1 | Scaffold | 99.36 | 1.91 | 5.30 | 45.6 |
|  |  | Mouse feces | A97 | GCA_910576255.1 | Scaffold | 99.36 | 1.91 | 5.30 | 45.6 |
|  |  | Mouse feces | MGBC103140 | GCA_910576745.1 | Scaffold | 97.78 | 0.00 | 5.00 | 46.0 |
|  |  | Human feces | DSM 935 | GCA_004340925.1 | Scaffold | 99.20 | 0.00 | 6.00 | 45.6 |
|  | *Blautia hydrogenotrophica* | Human feces | DSM 10507* | GCA_000157975.1 | Scaffold | 99.37 | 1.74 | 3.60 | 45.1 |
|  |  | Human feces | MGYG-HGUT-00035 | GCA_902362465.1 | Scaffold | 99.37 | 1.74 | 3.60 | 44.9 |
|  |  | Human feces | ERR1600647-bin.15 | GCA_905200475.1 | Contig | 98.35 | 1.58 | 3.20 | 45.5 |
|  |  | Human feces | 2789STDY5608857 | GCA_001404935.1 | Scaffold | 99.37 | 1.74 | 3.50 | 44.9 |
|  | *Blautia producta* | Human feces | DSM 2950* | GCA_014131715.1 | Complete | 99.36 | 0.00 | 6.25 | 45.7 |
|  |  | Human feces | MSK.4.17 | GCA_013302155.1 | Contig | 99.36 | 0.00 | 3.73 | 40.9 |
|  |  | Human feces | MSK.4.13 | GCA_013302185.1 | Contig | 99.36 | 0.00 | 3.73 | 40.9 |
|  |  | Sus scrofa feces | An915 | GCA_021531235.1 | Contig | 99.37 | 1.27 | 3.11 | 44.2 |
|  |  | Feces | PMF1 | GCA_004210255.1 | Complete | 99.36 | 0.21 | 6.35 | 45.6 |
|  |  | Human feces | I2DA | GCA_020697555.1 | Contig | 99.36 | 0.21 | 6.08 | 45.9 |
|  |  | Human feces | ID9B | GCA_020697595.1 | Contig | 98.81 | 0.21 | 5.98 | 46.0 |
|  |  | Human feces | ID8A | GCA_020697615.1 | Contig | 99.36 | 0.21 | 6.08 | 46.0 |
|  |  | Human feces | I24C | GCA_020697535.1 | Contig | 99.36 | 0.00 | 5.31 | 46.1 |
|  |  | Human feces | I31A | GCA_020697575.1 | Contig | 97.45 | 1.27 | 5.40 | 46.0 |
|  |  | Human feces | JCM 1471 | GCA_010669205.1 | Complete | 99.36 | 0.00 | 6.20 | 45.7 |
|  |  | Human gut | SRR5713945-bin.17 | GCA_905213955.1 | Contig | 94.90 | 0.00 | 5.75 | 46.9 |
|  |  | Human gut | ERR1430548-bin.7 | GCA_905202545.1 | Contig | 98.93 | 0.00 | 5.40 | 46.0 |
|  | *Blautia schinkii* | Human feces | MSK.6.32 | GCA_013302295.1 | Contig | 99.37 | 0.32 | 3.50 | 43.2 |
|  |  | Human feces | MSK.6.27 | GCA_013304875.1 | Contig | 99.37 | 0.32 | 3.70 | 43.2 |
|  |  | Human feces | MSK.14.32 | GCA_013304935.1 | Contig | 99.37 | 0.32 | 3.80 | 43.1 |
|  |  | Human feces | MSK.15.25 | GCA_013304885.1 | Contig | 99.37 | 0.32 | 3.80 | 43.1 |
|  |  | Human feces | MSK.6.16 | GCA_013304845.1 | Contig | 98.73 | 1.27 | 3.70 | 43.2 |
|  |  | Human feces | MSK.6.26 | GCA_013304825.1 | Contig | 99.37 | 0.32 | 3.50 | 43.2 |
|  |  | Human feces | MSK.6.25 | GCA_013304925.1 | Contig | 99.37 | 0.32 | 3.50 | 43.2 |
|  |  | Human feces | MSK.18.63 | GCA_020537945.1 | Contig | 99.37 | 0.00 | 4.10 | 42.9 |
|  |  | Rumen of suckling lamb | DSM 10518* | GCA_000702025.1 | Scaffold | 99.37 | 0.00 | 6.70 | 45.6 |
|  | *Clostridium autoethanogenum* | Rabbit feces | DSM 10061*** | GCA_001484725.1 | Complete | 98.62 | 0.50 | 4.35 | 31.1 |
|  | *Clostridium bovifaecis* | Cow manure | BXX*** | GCA_009738435.1 | Complete | 98.75 | 1.40 | 3.70 | 32.6 |
|  | *Clostridioides difficile* | Rumen, newborn lamb | BR81*** | GCA_002007885.1 | Complete | 99.46 | 0.53 | 4.12 | 28.7 |
|  |  | Patient gut | 630 (DSM 27543) | GCA_000932055.2 | Complete | 99.51 | 0.37 | 4.27 | 29.0 |
|  |  | Wolf | Z31 | GCA_001447175.1 | Complete | 96.33 | 0.63 | 4.30 | 29.2 |
|  |  | Sus scrofa feces | TW11 | GCA_009362915.1 | Complete | 97.04 | 0.86 | 4.14 | 29.1 |
|  |  | Human | DSM101085 | GCA_015732535.1 | Complete | 96.35 | 0.37 | 4.05 | 28.7 |
|  |  | Human | DSM29637 | GCA_003482255.1 | Complete | 99.51 | 0.37 | 4.11 | 28.8 |
|  |  | Human | DSM27639 | GCA_003313565.1 | Complete | 99.51 | 0.37 | 4.26 | 29.1 |
|  |  | Human | 020711 | GCA_003597915.1 | Complete | 98.02 | 0.62 | 4.09 | 28.5 |
|  |  | Human | M68 | GCA_000210395.1 | Complete | 99.51 | 0.56 | 4.31 | 28.9 |
|  |  | Human | ATCC9689 | GCA_001077535.2 | Complete | 97.25 | 0.78 | 4.15 | 28.7 |
|  |  | Human | DSM29747 | GCA_003482365.1 | Complete | 97.72 | 0.53 | 4.07 | 29.1 |
|  |  | Human | DSM28669 | GCA_003482125.1 | Complete | 99.35 | 0.76 | 4.14 | 28.8 |
|  |  | Human | DSM28666 | GCA_003482065.1 | Complete | 99.50 | 0.91 | 4.12 | 29.0 |
|  |  | Human feces | DSM105001 | GCA_014236775.1 | Complete | 99.51 | 0.37 | 4.20 | 28.8 |
|  | *Clostridium ljungdahlii* | Chicken yard waste | DSM 13528* | GCA_000143685.1 | Complete | 98.62 | 0.50 | 4.63 | 31.1 |
|  |  | Missing | ERI-2 | GCA_001636845.1 | Contig | 98.62 | 0.48 | 4.36 | 31.1 |
|  | *Eubacterium limosum* | Human intestinal content | ATCC8486*** | GCA_001636845.1 | Complete | 99.30 | 0.00 | 4.42 | 47.2 |
|  |  | Human feces | 81C1 | GCA_900683775.1 | Complete | 97.89 | 0.00 | 4.74 | 46.9 |
|  |  | rumen fluid | SA11 | GCA_001481725.1 | Complete | 99.30 | 0.00 | 4.15 | 47.4 |
|  |  | missing | 8486cho | GCA_003182515.1 | Scaffold | 99.30 | 0.00 | 4.37 | 47.2 |
|  |  | Human feces | DFI.6.107 | GCA_020559625.1 | Contig | 99.30 | 0.00 | 4.74 | 47.0 |
|  |  | Infant feces | L3_108_365G1_dasL3_108_365G1_concoct_69 | GCA_018363665.1 | Contig | 99.30 | 0.00 | 4.19 | 47.8 |
|  | *Eubacterium maltosivorans* | Human feces | Y1*** | GCA_002441855.2 | Complete | 99.30 | 0.00 | 4.34 | 47.8 |
|  |  | Human feces | BSD2780120874b_170522_C8 | GCA_015557125.1 | Scaffold | 99.30 | 0.70 | 4.17 | 47.6 |
|  |  | Human gut | MGYG-HGUT-00219 | GCA_902364445.2 | Scaffold | 99.30 | 0.00 | 4.24 | 47.6 |
|  |  | missing | 32_A2 | GCA_900103155.1 | Contig | 99.30 | 0.00 | 4.09 | 47.5 |
|  | *Marvinbryantia formatexigens* | Human feces | DSM 14469*** | GCA_000173815.1 | Contig | 98.73 | 0.16 | 4.55 | 49.6 |
|  |  | Human feces | I-52 | GCA_900102475.1 | Scaffold | 98.73 | 0.00 | 4.52 | 49.6 |
|  |  | Human gut | MGYG-HGUT-01698 | GCA_902377505.1 | Scaffold | 98.73 | 0.00 | 4.52 | 49.6 |
|  |  | Rat Cecum microbiota | S23_Bin3 | GCA_021771035.1 | Contig | 99.33 | 0.00 | 3.27 | 54.3 |
|  | *Moorella thermoacetica* | Horse manure | DSM 2955*** | GCA_001267435.1 | Complete | 99.23 | 0.00 | 2.62 | 55.8 |
|  |  | Horse faces | DSM 521 | GCA_001267405.1 | Complete | 99.23 | 0.00 | 2.53 | 55.9 |
|  |  | Horse faces | 39073-HH | GCA_006228565.1 | Complete | 99.23 | 0.00 | 2.65 | 55.8 |
|  | *Oxobacter pfennigii* | Steer rumen fluid | DSM 3222*** | GCA_001317355.1 | Contig | 99.19 | 4.03 | 4.51 | 39.0 |
|  | *Sporomusa termitida* | Wood-eating termite gut | DSM 4440*** | GCA_007641255.1 | Complete | 100.00 | 3.48 | 5.20 | 49.1 |
|  | *Terrisporobacter mayombei* | Soil-feeding termite gut | DSM 6539* | GCA_020748465.1 | Contig | 100.00 | 1.55 | 4.16 | 29.1 |
|  |  | Human feces | MSK.4.1 | GCA_020740325.1 | Contig | 100.00 | 1.64 | 4.06 | 28.9 |
|  | *Treponema primitia* | Termite hindgut | ZAS-2*** | GCA_000214375.1 | Complete | 98.86 | 1.14 | 4.06 | 50.8 |
|  |  | Termite hindgut | ZAS-1 | GCA_000297095.1 | Contig | 98.23 | 1.14 | 3.79 | 50.8 |
| **Non-gastrointestinal acetogens** | | | | | | | | | |
|  | *Acetoanaerobium noterae* | Sediment | ATCC 35199*** | GCA_900168025.1 | Scaffold | 98.60 | 1.40 | 2.81 | 33.4 |
|  | *Acetohalobium arabaticum* | Saline lagoon | DSM 5501* | GCA_000144695.1 | Complete | 97.41 | 0.00 | 2.47 | 36.6 |
|  | *Acetobacterium dehalogenans* | Sewage digester sludge | DSM 11527* | GCA_000472665.1 | Scaffold | 99.30 | 0.75 | 4.05 | 43.8 |
|  | *Acetobacterium malicum* | Ditch sediment | DER-2019*** | GCA_014284495.1 | Contig | 99.30 | 2.51 | 4.08 | 43.7 |
|  | *Acetobacterium paludosum* | Fen sediment | DSM 8237*** | GCA_008086595.1 | Contig | 99.30 | 3.05 | 3.69 | 40.0 |
|  |  | Bog sediment | DER-2019 | GCA_014284455.1 | Contig | 99.30 | 3.05 | 3.69 | 40.0 |
|  | *Acetobacterium tundrae* | Tundra wetland soil | DER-2019* | GCA_014284395.1 | Contig | 99.30 | 0.59 | 3.56 | 39.7 |
|  |  | Tundra soil | DSM 917 | GCA_008086615.1 | Contig | 99.30 | 0.59 | 3.56 | 39.6 |
|  | *Acetobacterium woodii* | Marine sediment | DSM 1030* | GCA_000247605.1 | Complete | 100.00 | 1.06 | 4.04 | 39.3 |
|  |  | Groundwater | NC_groundwater_1570_Pr3_B-0.1um_42_151 | GCA_016213825.1 | Contig | 99.30 | 1.10 | 3.94 | 41.6 |
|  | *Alkalibaculum bacchi* | Livestock-impacted soil | DSM 22112*** | GCA_003317055.1 | Scaffold | 98.59 | 0.70 | 3.10 | 34.7 |
|  |  | missing | DSM 221123 | GCA_008086585.1 | Contig | 98.59 | 0.70 | 3.11 | 34.7 |
|  | *Blautia producta* | Anaerobic digester sludge | DSM3507 | GCA_900107405.1 | Contig | 99.36 | 0.76 | 6.24 | 46.7 |
|  | *Carboxydothermus hydrogenoformans* | Hotspring | Z-2901*** | GCA_002915535.1 | Complete | 100.00 | 0.00 | 2.40 | 42.0 |
|  | *Clostridium aceticum* | Soil | DSM 1496*** | GCA_000012865.1 | Complete | 99.29 | 0.71 | 4.21 | 35.3 |
|  | *Clostridium autoethanogenum* | River sediment | H21-9 | GCA_000305935.1 | Contig | 98.62 | 0.15 | 4.61 | 32.8 |
|  | *Clostridium formicaceticum* | Sewage plant | DSM 92* | GCA_001042715.1 | Complete | 99.29 | 0.71 | 4.59 | 35.5 |
|  |  | Sewage plant | ATCC 27076 | GCA_001038625.1 | Complete | 99.29 | 0.71 | 4.59 | 35.5 |
|  | *Lacrimispora indolis* | Olive oil mill wastewater | SR3*** | GCA_001854185.1 | Contig | 99.37 | 0.95 | 7.09 | 44.5 |
|  | *Clostridium scatologenes* | Soil | ATCC 25775*** | GCA_900129955.1 | Complete | 100  .00 | 3.35 | 5.75 | 29.6 |
|  | *Clostridium ultunense* | Swine manure digester | DSM 10521*** | GCA_000421505.1 | Scaffold | 99.30 | 0.35 | 3.22 | 32.9 |
|  | *Eubacterium aggregans* | Olive oil mill wastewater | SR12*** | GCA_000511955.1 | Contig | 99.30 | 0.00 | 2.83 | 48.6 |
|  | *Holophaga foetida* | Black anoxic freshwater mud | DSM 6591* | GCA_900120235.1 | Scaffold | 99.12 | 0.88 | 4.13 | 62.9 |
|  | *Moorella glycerini* | Hot spring sediment | DSM 11254*** | GCA_900107815.1 | Complete | 96.94 | 1.02 | 3.56 | 54.7 |
|  |  | Underground gas storage site | NMP | GCA_000242615.3 | Scaffold | 98.98 | 3.83 | 3.58 | 53.8 |
|  | *Moorella mulderi* | Thermophilic bioreactor | DSM 14980*** | GCA_009735625.1 | Contig | 98.81 | 0.68 | 3.00 | 54.5 |
|  | *Moorella thermoacetica* | Mud and water from hot spring | DSM 103284 | GCA_001508215.1 | Complete | 98.98 | 0.64 | 2.56 | 55.9 |
|  |  | Mud and water from hot spring | DSM 103132 | GCA_000763575.1 | Complete | 99.23 | 1.83 | 2.98 | 55.1 |
|  | *Sporomusa malonica* | Freshwater sediment | DSM 5090*** | GCA_001874605.1 | Scaffold | 99.91 | 3.09 | 5.19 | 44.5 |
|  | *Sporomusa silvacetica* | Forest soil | DSM 10669*** | GCA_900101845.1 | Contig | 100.00 | 2.66 | 5.93 | 43.0 |
|  | *Sporomusa sphaeroides* | River mud | DSM 2875*** | GCA_001941975.1 | Contig | 100.00 | 3.69 | 4.97 | 47.2 |
|  | *Terrisporobacter glycolicus* | Mud | ATCC 14880* | GCA_000423685.1 | Scaffold | 100.00 | 0.00 | 4.00 | 28.4 |
|  |  | Dairy farm | FS03 | GCA_002257705.1 | Contig | 100.00 | 0.00 | 3.95 | 28.6 |
|  |  | Missing | KPPR-9 | GCA_001941975.1 | Scaffold | 100.00 | 0.00 | 3.99 | 28.6 |
|  |  | Waste Water | WW3900 | GCA_000373865.1 | Contig | 100.00 | 0.00 | 3.88 | 28.6 |
|  |  | Human gut | MGYG-HGUT-00005 | GCA_003508495.1 | Scaffold | 99.30 | 0.00 | 3.93 | 28.6 |
|  | *Tindallia californiensis* | Mono Lake | APO*** | GCA_017307015.1 | Scaffold | 98.60 | 0.00 | 3.12 | 39.8 |
|  | *Thermacetogenium phaeum* | Pulp waste water reactor | DSM 12270*** | GCA_900114105.1 | Complete | 98.52 | 0.00 | 2.94 | 53.9 |
|  | *Thermoanaerobacter kivui* | Marine sediment | LKT-1*** | GCA_902362305.1 | Complete | 98.93 | 2.71 | 2.40 | 35.0 |

*, Genome sequences analyzed in pangenome, phylogenetic and positive pressure analysis of this study are indicated in asterisk (*).

**Table S2** Units and copy numbers of genes encoding enzymes involved in Wood-Ljungdahl pathway in the genomes of 43 acetogen species

| **No** | **Species** | **Strain** | **Gene copy number** | | | | | | | | | | |
| --- | --- | --- | --- | --- | --- | --- | --- | --- | --- | --- | --- | --- | --- |
|  |  |  | ***fdhF*** | ***fhs*** | ***folD*** | ***fchA*** | ***metF*** | ***metV*** | ***acsA*** | ***acsB*** | ***acsE*** | ***acsD*** | ***acsC*** |
| **Gastrointestinal acetogens** | | | | | | | | | | | | | |
|  | *Acetobacterium fimetarium* | DER-2019*** | 2 | 1 | 1 | 1 | 1 | 1 | 1 | 2 | 6 | 1 | 1 |
|  | *Acetonema longum* | DSM 6540*** | 1 | 1 | 1 | 1 | 0 | 1 | 3 | 1 | 3 | 1 | 3 |
|  | *Blautia coccoides* | NCTC11035*** | 1 | 1 | 1 | 2 | 1 | 0 | 3 | 1 | 1 | 1 | 1 |
|  |  | A1_1.BP.CDM | 1 | 1 | 1 | 2 | 1 | 0 | 3 | 1 | 1 | 1 | 1 |
|  |  | A97 | 1 | 1 | 1 | 2 | 1 | 0 | 3 | 1 | 1 | 1 | 1 |
|  |  | MGBC103140 | 1 | 1 | 1 | 2 | 1 | 0 | 3 | 1 | 1 | 1 | 1 |
|  |  | DSM 935 | 1 | 1 | 1 | 2 | 1 | 0 | 3 | 1 | 1 | 1 | 1 |
|  | *Blautia hydrogenotrophica* | DSM 10507* | 1 | 1 | 1 | 1 | 1 | 1 | 3 | 3 | 1 | 0 | 1 |
|  |  | MGYG-HGUT-00035 | 1 | 1 | 1 | 1 | 1 | 1 | 3 | 3 | 1 | 1 | 1 |
|  |  | ERR1600647-bin.15 | 1 | 1 | 1 | 1 | 1 | 1 | 3 | 3 | 1 | 1 | 1 |
|  |  | 2789STDY5608857 | 1 | 1 | 1 | 1 | 1 | 1 | 3 | 3 | 1 | 1 | 1 |
|  | *Blautia producta* | DSM 2950* | 1 | 1 | 1 | 2 | 1 | 0 | 3 | 1 | 4 | 1 | 1 |
|  |  | MSK.4.17 | 0 | 2 | 1 | 2 | 1 | 0 | 1 | 2 | 1 | 1 | 1 |
|  |  | MSK.4.13 | 0 | 2 | 1 | 2 | 1 | 0 | 1 | 2 | 1 | 1 | 1 |
|  |  | An915 | 0 | 1 | 1 | 1 | 1 | 1 | 1 | 1 | 2 | 1 | 1 |
|  |  | PMF1 | 1 | 1 | 1 | 2 | 1 | 0 | 1 | 3 | 4 | 1 | 1 |
|  |  | I2DA | 0 | 1 | 1 | 2 | 1 | 1 | 4 | 3 | 4 | 1 | 1 |
|  |  | ID9B | 0 | 1 | 1 | 2 | 1 | 1 | 4 | 3 | 4 | 1 | 1 |
|  |  | ID8A | 0 | 1 | 1 | 2 | 1 | 1 | 4 | 3 | 4 | 1 | 1 |
|  |  | I24C | 0 | 1 | 1 | 2 | 1 | 1 | 2 | 3 | 4 | 1 | 1 |
|  |  | I31A | 0 | 1 | 1 | 2 | 1 | 1 | 2 | 3 | 4 | 1 | 1 |
|  |  | JCM 1471 | 1 | 1 | 1 | 2 | 1 | 0 | 1 | 3 | 4 | 1 | 1 |
|  |  | SRR5713945-bin.17 | 0 | 1 | 1 | 2 | 1 | 1 | 4 | 3 | 4 | 1 | 1 |
|  |  | ERR1430548-bin.7 | 0 | 1 | 1 | 2 | 1 | 1 | 4 | 3 | 4 | 1 | 1 |
|  | *Blautia schinkii* | MSK.6.32 | 0 | 2 | 1 | 1 | 2 | 1 | 1 | 0 | 5 | 1 | 1 |
|  |  | MSK.6.27 | 0 | 2 | 1 | 1 | 2 | 1 | 1 | 0 | 3 | 1 | 1 |
|  |  | MSK.14.32 | 0 | 2 | 1 | 1 | 2 | 1 | 1 | 0 | 3 | 1 | 1 |
|  |  | MSK.15.25 | 0 | 2 | 1 | 1 | 2 | 1 | 1 | 0 | 3 | 1 | 1 |
|  |  | MSK.6.16 | 0 | 2 | 1 | 1 | 2 | 1 | 1 | 0 | 4 | 1 | 1 |
|  |  | MSK.6.26 | 0 | 2 | 1 | 1 | 2 | 1 | 1 | 0 | 4 | 1 | 1 |
|  |  | MSK.6.25 | 0 | 2 | 1 | 1 | 2 | 1 | 1 | 0 | 4 | 1 | 1 |
|  |  | MSK.18.63 | 0 | 2 | 1 | 1 | 2 | 1 | 1 | 2 | 2 | 1 | 1 |
|  |  | DSM 10518* | 1 | 2 | 1 | 1 | 2 | 1 | 1 | 2 | 4 | 1 | 1 |
|  | *Clostridium autoethanogenum* | DSM 10061*** | 3 | 1 | 1 | 3 | 1 | 1 | 3 | 2 | 5 | 1 | 1 |
|  | *Clostridium bovifaecis* | BXX*** | 0 | 1 | 1 | 1 | 1 | 0 | 2 | 0 | 1 | 0 | 1 |
|  | *Clostridioides difficile* | BR81*** | 1 | 1 | 1 | 1 | 1 | 1 | 2 | 1 | 1 | 1 | 1 |
|  |  | 630 (DSM 27543) | 0 | 1 | 1 | 1 | 1 | 1 | 2 | 1 | 1 | 1 | 1 |
|  |  | Z31 | 0 | 1 | 0 | 1 | 1 | 1 | 3 | 1 | 1 | 1 | 1 |
|  |  | TW11 | 1 | 1 | 1 | 1 | 1 | 1 | 2 | 1 | 3 | 1 | 1 |
|  |  | DSM101085 | 1 | 1 | 1 | 1 | 1 | 1 | 2 | 1 | 2 | 1 | 1 |
|  |  | DSM29637 | 0 | 1 | 1 | 1 | 1 | 1 | 2 | 1 | 2 | 1 | 1 |
|  |  | DSM27639 | 0 | 1 | 1 | 1 | 1 | 1 | 2 | 1 | 4 | 1 | 1 |
|  |  | 020711 | 0 | 1 | 1 | 1 | 1 | 1 | 2 | 1 | 2 | 1 | 1 |
|  |  | M68 | 0 | 1 | 1 | 1 | 1 | 1 | 2 | 1 | 2 | 1 | 1 |
|  |  | ATCC9689 | 1 | 1 | 1 | 1 | 1 | 1 | 2 | 1 | 2 | 1 | 1 |
|  |  | DSM29747 | 1 | 1 | 1 | 1 | 1 | 1 | 2 | 1 | 2 | 1 | 1 |
|  |  | DSM28669 | 1 | 1 | 1 | 1 | 1 | 1 | 2 | 1 | 2 | 1 | 1 |
|  |  | DSM28666 | 1 | 1 | 1 | 1 | 1 | 1 | 2 | 1 | 2 | 1 | 1 |
|  |  | DSM105001 | 1 | 1 | 1 | 1 | 1 | 1 | 2 | 1 | 3 | 1 | 1 |
|  | *Clostridium ljungdahlii* | DSM 13528* | 1 | 1 | 1 | 3 | 1 | 1 | 4 | 1 | 8 | 1 | 1 |
|  |  | ERI-2 | 3 | 1 | 1 | 2 | 1 | 1 | 3 | 3 | 6 | 1 | 1 |
|  | *Eubacterium limosum* | ATCC8486*** | 1 | 1 | 1 | 1 | 2 | 1 | 1 | 1 | 5 | 1 | 1 |
|  |  | 81C1 | 1 | 1 | 1 | 1 | 3 | 1 | 1 | 1 | 6 | 1 | 1 |
|  |  | SA11 | 1 | 1 | 1 | 1 | 2 | 1 | 1 | 1 | 5 | 1 | 1 |
|  |  | 8486cho | 1 | 1 | 1 | 1 | 2 | 1 | 1 | 1 | 7 | 1 | 1 |
|  |  | DFI.6.107 | 1 | 1 | 1 | 1 | 2 | 1 | 1 | 1 | 8 | 1 | 1 |
|  |  | L3_108_365G1_dasL3_108_365G1_concoct_69 | 1 | 1 | 1 | 1 | 2 | 1 | 1 | 1 | 9 | 1 | 1 |
|  | *Eubacterium maltosivorans* | Y1*** | 1 | 1 | 1 | 1 | 2 | 1 | 1 | 3 | 5 | 1 | 1 |
|  |  | BSD2780120874b_170522_C8 | 1 | 1 | 1 | 1 | 2 | 1 | 1 | 1 | 7 | 1 | 1 |
|  |  | MGYG-HGUT-00219 | 1 | 1 | 1 | 1 | 2 | 1 | 1 | 1 | 5 | 1 | 1 |
|  |  | 32_A2 | 1 | 1 | 1 | 1 | 2 | 1 | 1 | 1 | 6 | 1 | 1 |
|  | *Marvinbryantia formatexigens* | DSM 14469*** | 0 | 2 | 1 | 1 | 1 | 1 | 2 | 1 | 3 | 1 | 1 |
|  |  | I-52 | 0 | 2 | 1 | 1 | 1 | 1 | 2 | 1 | 2 | 1 | 1 |
|  |  | MGYG-HGUT-01698 | 0 | 2 | 1 | 1 | 1 | 1 | 2 | 1 | 3 | 1 | 1 |
|  |  | S23_Bin3 | 0 | 2 | 1 | 1 | 1 | 1 | 3 | 1 | 3 | 1 | 1 |
|  | *Moorella thermoacetica* | DSM 2955*** | 2 | 1 | 1 | 0 | 1 | 1 | 2 | 1 | 1 | 1 | 1 |
|  |  | DSM 521 | 2 | 1 | 1 | 0 | 1 | 1 | 2 | 1 | 5 | 1 | 1 |
|  |  | 39073-HH | 1 | 1 | 1 | 0 | 1 | 1 | 2 | 1 | 5 | 1 | 1 |
|  | *Oxobacter pfennigii* | DSM 3222*** | 3 | 1 | 3 | 1 | 1 | 3 | 3 | 2 | 5 | 1 | 1 |
|  | *Sporomusa termitida* | DSM 4440*** | 2 | 4 | 1 | 3 | 2 | 2 | 4 | 1 | 5 | 1 | 1 |
|  | *Terrisporobacter mayombei* | DSM 6539* | 1 | 2 | 2 | 2 | 1 | 1 | 2 | 1 | 1 | 1 | 1 |
|  |  | MSK.4.1 | 2 | 2 | 2 | 2 | 1 | 1 | 2 | 1 | 2 | 1 | 1 |
|  | *Treponema primitia* | ZAS-2*** | 2 | 1 | 1 | 1 | 1 | 0 | 1 | 0 | 3 | 1 | 1 |
|  |  | ZAS-1 | 1 | 1 | 1 | 2 | 1 | 0 | 2 | 1 | 3 | 1 | 1 |
| **Non-gastrointestinal acetogens** | | | | | | | | | | | | | |
|  | *Acetoanaerobium noterae* | ATCC 35199*** | 1 | 1 | 1 | 1 | 1 | 1 | 1 | 1 | 1 | 1 | 1 |
|  | *Acetohalobium arabaticum* | DSM 5501* | 1 | 1 | 1 | 1 | 0 | 1 | 2 | 4 | 4 | 1 | 1 |
|  | *Acetobacterium dehalogenans* | DSM 11527* | 2 | 1 | 1 | 1 | 1 | 1 | 2 | 3 | 17 | 1 | 1 |
|  | *Acetobacterium malicum* | DER-2019*** | 1 | 1 | 0 | 1 | 1 | 1 | 2 | 3 | 12 | 1 | 1 |
|  | *Acetobacterium paludosum* | DSM 8237*** | 2 | 2 | 1 | 1 | 2 | 1 | 2 | 2 | 8 | 1 | 1 |
|  |  | DER-2019 | 2 | 2 | 1 | 1 | 2 | 1 | 2 | 2 | 7 | 1 | 1 |
|  | *Acetobacterium tundrae* | DER-2019* | 2 | 1 | 1 | 1 | 1 | 1 | 2 | 2 | 5 | 1 | 1 |
|  |  | DSM 917 | 2 | 1 | 1 | 1 | 1 | 1 | 2 | 2 | 6 | 1 | 1 |
|  | *Acetobacterium woodii* | DSM 1030* | 2 | 2 | 1 | 1 | 1 | 1 | 1 | 1 | 8 | 1 | 1 |
|  |  | NC_groundwater_1570_Pr3_B-0.1um_42_151 | 2 | 2 | 1 | 1 | 1 | 1 | 3 | 3 | 8 | 1 | 1 |
|  | *Alkalibaculum bacchi* | DSM 22112*** | 2 | 1 | 1 | 3 | 2 | 2 | 3 | 1 | 3 | 1 | 1 |
|  |  | DSM 221123 | 2 | 1 | 1 | 3 | 2 | 2 | 3 | 1 | 5 | 1 | 1 |
|  | *Blautia producta* | DSM3507 | 1 | 1 | 1 | 2 | 1 | 0 | 2 | 1 | 2 | 1 | 1 |
|  | *Carboxydothermus hydrogenoformans* | Z-2901*** | 1 | 1 | 1 | 2 | 1 | 0 | 3 | 4 | 1 | 1 | 1 |
|  | *Clostridium aceticum* | DSM 1496*** | 1 | 1 | 1 | 2 | 2 | 1 | 4 | 1 | 3 | 1 | 1 |
|  | *Clostridium autoethanogenum* | H21-9 | 1 | 1 | 1 | 2 | 1 | 1 | 3 | 1 | 3 | 1 | 1 |
|  | *Clostridium formicaceticum* | DSM 92* | 2 | 1 | 1 | 2 | 2 | 1 | 5 | 2 | 4 | 1 | 1 |
|  |  | ATCC 27076 | 2 | 1 | 1 | 2 | 2 | 1 | 5 | 1 | 3 | 1 | 1 |
|  | *Lacrimispora indolis* | SR3*** | 1 | 1 | 1 | 1 | 2 | 0 | 1 | 0 | 1 | 0 | 0 |
|  | *Clostridium scatologenes* | ATCC 25775*** | 2 | 2 | 1 | 2 | 2 | 1 | 3 | 3 | 3 | 1 | 1 |
|  | *Clostridium ultunense* | DSM 10521*** | 1 | 1 | 1 | 1 | 0 | 0 | 1 | 0 | 0 | 0 | 0 |
|  | *Eubacterium aggregans* | SR12*** | 1 | 1 | 1 | 1 | 1 | 1 | 1 | 1 | 4 | 1 | 1 |
|  | *Holophaga foetida* | DSM 6591* | 1 | 1 | 1 | 0 | 1 | 1 | 1 | 1 | 2 | 1 | 1 |
|  | *Moorella glycerini* | DSM 11254*** | 1 | 1 | 1 | 0 | 1 | 1 | 3 | 3 | 3 | 1 | 1 |
|  |  | NMP | 1 | 1 | 1 | 0 | 2 | 2 | 3 | 2 | 4 | 2 | 2 |
|  | *Moorella mulderi* | DSM 14980*** | 1 | 1 | 1 | 1 | 1 | 1 | 4 | 1 | 1 | 1 | 1 |
|  | *Moorella thermoacetica* | DSM 103284 | 2 | 1 | 1 | 0 | 1 | 1 | 2 | 1 | 5 | 1 | 1 |
|  |  | DSM 103132 | 2 | 1 | 1 | 0 | 1 | 1 | 2 | 1 | 4 | 1 | 1 |
|  | *Sporomusa malonica* | DSM 5090*** | 1 | 2 | 1 | 5 | 2 | 2 | 3 | 2 | 8 | 1 | 1 |
|  | *Sporomusa silvacetica* | DSM 10669*** | 2 | 2 | 1 | 5 | 2 | 2 | 3 | 2 | 6 | 1 | 1 |
|  | *Sporomusa sphaeroides* | DSM 2875*** | 3 | 2 | 1 | 5 | 2 | 2 | 3 | 2 | 8 | 1 | 1 |
|  | *Terrisporobacter glycolicus* | ATCC 14880* | 1 | 2 | 2 | 2 | 2 | 1 | 3 | 1 | 1 | 1 | 1 |
|  |  | FS03 | 1 | 2 | 2 | 3 | 1 | 1 | 3 | 0 | 1 | 1 | 1 |
|  |  | KPPR-9 | 1 | 2 | 2 | 3 | 1 | 1 | 3 | 1 | 1 | 1 | 1 |
|  |  | WW3900 | 1 | 2 | 2 | 3 | 1 | 1 | 2 | 1 | 2 | 1 | 1 |
|  |  | MGYG-HGUT-00005 | 1 | 2 | 2 | 3 | 1 | 1 | 2 | 1 | 3 | 1 | 1 |
|  | *Tindallia californiensis* | APO*** | 2 | 1 | 1 | 1 | 1 | 1 | 0 | 0 | 1 | 0 | 0 |
|  | *Thermacetogenium phaeum* | DSM 12270*** | 3 | 2 | 2 | 2 | 1 | 1 | 2 | 2 | 3 | 1 | 1 |
|  | *Thermoanaerobacter kivui* | LKT-1*** | 1 | 1 | 1 | 2 | 1 | 1 | 1 | 1 | 1 | 1 | 1 |

*, Genome sequences analyzed in pangenome, phylogenetic and positive pressure analysis of this study are indicated in asterisk (*).

**Table** S3 Likelihood ratio tests of the site model M1a vs M2a for *acsA*, *acsB*, *acsC*, *acsD*, *fdhF*, *fhs*, *fchA*, *folD*, *metF*, *metV*, *ascE* of acetogens. Abbreviations: number of parameters (np), likelihood value (ln L), omega value (ω).

| Gene | Model | | np | （ln *L*） | 2 Δln *L* | *P* |
| --- | --- | --- | --- | --- | --- | --- |
| *fdhF* | | M1a | 88 | -92328.3 | 5.8000 | 0.0550 |
|  |  | M2a | 90 | -92325.4 |  |  |
| *fhs* | | M1a | 96 | -41876.2 | 0 | 1 |
|  |  | M2a | 98 | -41876.2 |  |  |
| *fchA* | | M1a | 90 | -16122.3 | 0 | 1 |
|  |  | M2a | 92 | -16122.3 |  |  |
| *folD* | | M1a | 90 | -24325.7 | 0 | 1 |
|  |  | M2a | 92 | -24325.7 |  |  |
| *metF* | | M1a | 86 | -21222.5 | 0 | 1 |
|  |  | M2a | 88 | -21222.5 |  |  |
| *metV* | | M1a | 80 | -16178.2 | 0 | 1 |
|  |  | M2a | 82 | -16178.2 |  |  |
| *ascE* | | M1a | 90 | -20233.6 | 0 | 1 |
|  |  | M2a | 92 | -20233.6 |  |  |
| *acsB* | | M1a | 94 | -71256.3 | 0 | 1 |
|  |  | M2a | 96 | -71256.3 |  |  |
| *acsA* | | M1a | 86 | -27893.2 | 0 | 1 |
|  |  | M2a | 88 | -27893.2 |  |  |
| *acsC* | | M1a | 88 | -24653.9 | 0 | 1 |
|  |  | M2a | 90 | -24653.9 |  |  |
| *acsD* | | M1a | 86 | -22653.6 | 0 | 1 |
|  |  | M2a | 88 | -22653.6 |  |  |

**Supplemental Figures (S1)**


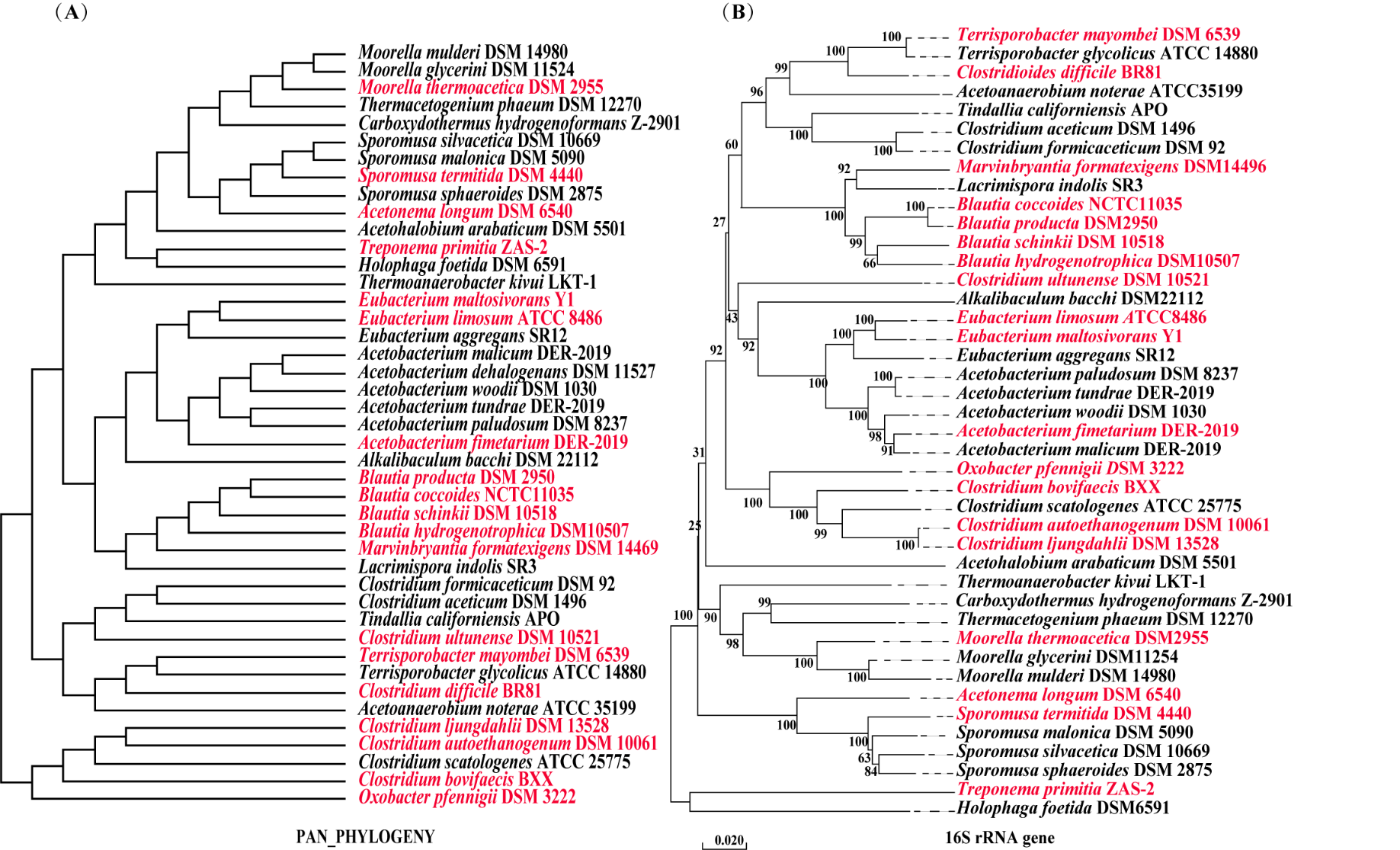


**Fig. S1** Neighbor-joining phylogenetic tree based on pan-genome (A) and 16S rRNA genes (B) of the 43 acetogen species. Red and black fonts represent gastrointestinal and non-gastrointestinal acetogens, respectively
